# LGI-1 encephalopathy following ChAdOx1 nCov-19 vaccination

**DOI:** 10.1186/s42466-022-00187-8

**Published:** 2022-06-20

**Authors:** Tamara Garibashvili, Josef Georg Heckmann

**Affiliations:** Department of Neurology, Municipal Hospital Landshut, Robert-Koch Str. 1, 84034 Landshut, Germany

**Keywords:** LGI1 encephalitis, ChAdOx1 nCov-19 vaccination, Covid-19

## Abstract

A 71-year-old male patient was diagnosed with LGI1 encephalopathy 4 weeks following a first ChAdOx1 nCov-19 vaccination. Extensive work-up including analysis of CSF and PET examination did not reveal a tangible cause so that a vaccine-associated encephalopathy was considered as differential diagnosis. Under steroid treatment, the faciobrachial dystonic seizures subsided.

Dear Sir,

We read with great interest the report by Walter and Kraemer [1] on a neurologist’s rhombencephalitis after comirnaty vaccination, in which a possible relationship between the vaccination and this neuroinflammatory disease was discussed. Equally worthy of consideration are the critical remarks regarding the possibility of the causal connection, so that the debate about a coincidental or causal connection continues [2]. Furthermore, we were alerted to reports of post-COVID-19 opsoclonus-myoclonus syndrome and encephalopathy associated with leucine-rich glioma-inactivated 1 (LGI-1) antibodies [3] and anti-LGI1 encephalitis following COVID-19 vaccination [4]. We would like to contribute to this issue with a case of LGI-1 encephalopathy following vaccination with the ChAdOx1 nCov-19 vaccine in which we discuss a possible connection to vaccination.

Four weeks after his first ChAdOx1 nCov-19 vaccination a 71-year-old patient developed cardiac pauses necessitating a pacemaker. A pre-existing heart disease could be ruled out by medical examination. Only a few days later he suffered left faciobrachial dystonic seizures. These episodes lasted 2–3 s and occurred 2–5 times a day. Clinically, the patient was awake, fully oriented and showed a normal neurological and cognitive status aside mild pallhypaethesia. During the clinical examination, several separate faciobrachial dystonic seizures were observed. The medical history otherwise revealed arterial hypertension and hyperlipidemia. Cranial computed tomography showed no abnormalities. Electroencephalography was without epileptic potentials. Cerebrospinal fluid (CSF) analysis was normal. Immunological diagnostics demonstrated a marked increase in LG1 antibodies in the serum of 1:160 (normal value < 1:10). These antibodies were not elevated in the CSF. The other autoantibodies, investigated as suggested by Bien and Bien [5], CASPR-2, DPPX, GABA, AMPA 1/2, glutamate receptor, glycine receptor, Hu, amphyphysin, CV2, Da and MOG were negative or normal in both CSF and serum. Whole-body PET showed no evidence of FDG-positive nuclide accumulation. Under the working diagnosis of an LG1-positive autoimmune encephalopathy, a steroid pulse therapy was given which rapidly led to the cessation of the faciobrachial dystonic seizures. Currently, the patient is on oral steroid therapy (25 mg prednisolone) and has remained hitherto over six months’ seizure-free. At this time the LG1 autoantibodies are still subtly detectable in the serum (1:20; normal value < 1:10).

In our case, we made the diagnosis of autoimmune encephalopathy based on the Graus criteria [6] including subacute onset, new focal CNS findings (cardiac pauses, facio-brachio-dystonic seizures), evidence of LGI1 autoantibodies and reasonable exclusion of alternative causes. In addition, we consider a causal relationship with the preceding vaccination as the symptomatology manifested itself within the critical 6 weeks following vaccination with the first ChAdOx1 nCov-19 dose and since no alternative cause could be found in the detailed additional examinations. The symptomatology with cardiac pauses and isolated faciobrachial dystonic seizures without cognitive impairment and without prolonged preexisting abnormalities also suggests a relatively acute onset of disease rather than a slowly creeping progression with cognitive decline as is otherwise typical of autoimmune LGI1-encephalitis [7]. Moreover, normal MRI findings and normal results of standard CSF studies as in our patient are frequent in encephalopathy with LGI1 antibodies [8]. The cardiac pauses, that required a pacemaker in our patient, are a rare but known consequence of autonomic instability in autoimmune encephalopathy [8].

Zuhorn et al. [9] recently reported on 3 cases of postvaccinal autoimmune encephalitis following ChAdOx1 nCov-19 vaccination. In their additional analysis of the publicly available database, they estimated the incidence of such a possible association to be 0.1 per 100,000 vaccinations for the ChAdOx1 nCov-19 and 0.04 per 100,000 vaccinations for the mRNA vaccine (Comirnaty) [9]. It appears that SARS-CoV2 itself as well as adverse reactions to SARS-CoV2 vaccinations show a certain tropism for neuronal structures and tissues inducing neurological side effects [10]. Simplistically, given that ChAdOx1 nCov-19 can result in platelet-activating antibodies against PF4 [11], it is quite possible that antibodies against other structures such as LGI1 antibodies can be produced, resulting in encephalopathy as in our patient. Nevertheless, a coincidental occurrence between the LGI1 encephalopathy in our patient and the previous vaccination cannot be ruled out, and we must await further observations, as it is proposed regarding associations between Covid-19 vaccine and the Guillain-Barré syndrome [12].

## Conclusion

LGI1 encephalopathy can possibly occur in rare cases after SARS Cov2 vaccination.

## Data Availability

The main data analyzed in this case vignette are included in the manuscript.
